# N6-Methyladenosine Modification of the Three Components “Writers”, “Erasers”, and “Readers” in Relation to Osteogenesis

**DOI:** 10.3390/ijms26125620

**Published:** 2025-06-12

**Authors:** Qiannan Dong, Xubin Zhao, Changze Zhu, Jianping Ruan, Cheng Chen

**Affiliations:** 1 Key Laboratory of Shaanxi Province for Craniofacial Precision Medicine Research, Xi’an 710000, China; 2 Department of General Dentistry, College of Stomatology, Xi’an Jiaotong University, Xi’an 710000, China; 3 Center of Oral Public Health, College of Stomatology, Xi’an Jiaotong University, Xi’an 710000, China

**Keywords:** osteoporosis, inflammation, osteogenic differentiation, bone regeneration, N6-methyladenosine, epigenetic modification

## Abstract

Bone-related diseases significantly diminish human happiness, adversely impacting overall quality of life. Optimizing bone tissue repair remains a prominent focus within the field of bone tissue regenerative medicine. N6-methyladenosine (m6A) is one of the most prevalent epigenetic modifications found in eukaryotic mRNA and non-coding RNA. The functions of m6A involve diverse components, including “Writers”, “Erasers”, and “Readers”. Numerous studies have demonstrated that m6A plays a crucial role in the exchange of information and coordination among various cell types, bioactive factors, and the microenvironment, influencing the progression of diverse physiological and pathological processes within the human body. In recent years, many functions and molecular pathways associated with m6A have been identified. This review primarily discusses the relationship between the three components of m6A and osteogenesis, as well as other key genes and pathways involved in this process. Additionally, we provide an in-depth elucidation of the interaction network between m6A modifications, micro-RNAs, and long non-coding RNAs. In the final section, we address the current limitations in m6A and osteogenesis research and explore the prospects for the diagnosis and treatment of bone-related diseases.

## 1. Introduction

Bone significantly contributes to the quality of daily human life. It is a highly adaptable and dynamic tissue [1]. Bone homeostasis is influenced by a range of factors, which include the exchange of information and coordination among diverse cell types, various bioactive factors, a multitude of hormones, and the bone microenvironment, all of which work together to continuously reshape and maintain the integrity of bone structure [2]. When bone homeostasis is disrupted, insufficient bone formation, excessive bone resorption, or excessive conversion of fat can lead to bone loss and even bone defects [3]. Nowadays, due to the increasing prevalence of aging, bone-related diseases have resulted in substantial financial and medical burdens due to their high incidence, disability rates, and mortality, presenting serious challenges and making them a critical public health concern that warrants attention and resolution [4,5,6].

RNA methylation is a significant epigenetic regulatory process in eukaryotes, occurring in various RNAs [7,8]. This process manifests in various forms, including N6-methyladenosine (m6A), N1-methyladenosine (m1A) [9], 5-methylcytosine (m5C) [10], and 7-methylguanine (m7G) [11]. It plays a crucial role in numerous physiological and pathological processes within the human body [7]. Research has indicated that dysmethylation of RNA is a key factor contributing to the dysfunction associated with various human diseases [12,13].

**Figure 1:**
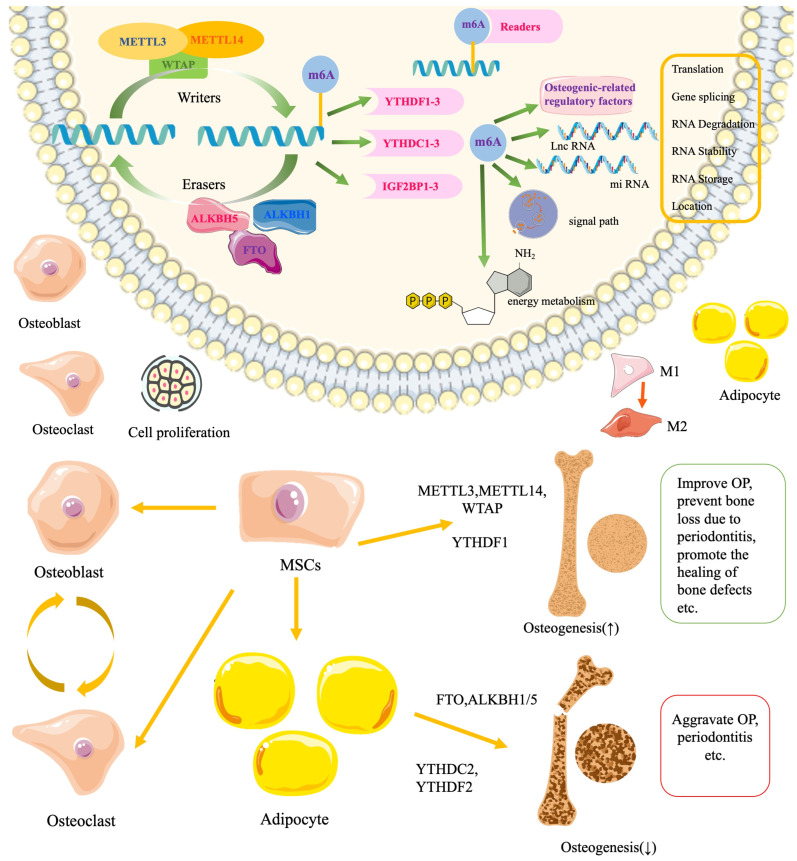
The roles of three elements of m6A methylation, “Writer”, “Eraser”, and “Reader”, in RNA epigenetic modification and m6A methylation modification involved in osteogenic biological processes through various means.

**Figure 2:**
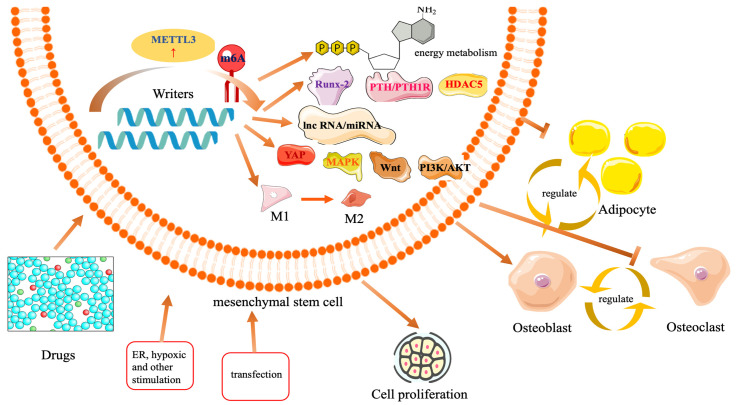
Multiple stimuli, such as drugs, endoplasmic reticulum stress, hypoxia stimulation, etc., all lead to an increase in METTL3 expression in mesenchymal stem cells, as well as an increase in m6A methylation modification. They regulate various osteogenic regulatory transcription factors, lncRNAs/miRNAs, and signaling pathways to exert anti-inflammatory effects, promote cell proliferation, and regulate osteogenic differentiation, osteoclast genesis, and the conversion of osteoblasts to adipocytes.

m6A is one of the most common and abundant mRNA methylation modification modes, affecting various types of RNA, including mRNA, tRNA, rRNA, circular RNA (circRNA), miRNA, and long non-coding RNA (lncRNA), responsible for regulating gene splicing, transport, stability, storage, localization, translation state, and the formation of higher-order structures. This modification can affect gene expression without altering the nucleotide sequence, representing a reversible change driven by chemical modification [14]. It is regulated by methyltransferases, demethylases, and methyl-reading proteins, which are responsible for methylation, demethylation, and decoding the methylation code [15,16]. Methyltransferases are enzymes that facilitate the methylation of bases on mRNA, specifically targeting the N6 amino group of adenines. They catalyze RNA methylation both in vivo and in vitro, with notable examples including methyltransferase-like proteins 3, 14, and 16 (METTL3/14/16), Wilms’ tumor 1-associated protein (WTAP), and RNA-binding motif proteins 15 and 15B. Conversely, demethylases are proteins that lower m6A levels by diminishing the activity of oxidative demethylases, converting the methyl group of m6A back to adenosine, and mediating the demethylation process. Key demethylases include ALKB homolog 5 (ALKBH5), an α-ketoglutarate-dependent dioxygenase, and fat mass and obesity-associated protein (FTO). The methylated reading protein is crucial for recognizing the m6A modification and translating it into various functional signals. It plays a significant role in downstream processes, including mRNA variable splicing, nuclear export, translation, degradation, maintenance of mRNA stability, and miRNA processing. Key proteins involved in these processes include YT521-B homology (YTH) domain-containing proteins 1 and 2 (YTHDC1/2), YTH N6-methyladenosine RNA-binding proteins 1, 2, and 3 (YTHN6), YTHDF proteins 1, 2, and 3 (YTHDF1/2/3), and IGF2 mRNA-binding proteins 1, 2, and 3 (IGF2BP1/2/3). The balance of m6A levels is maintained through the interaction between methyltransferases and demethyltransferases [17,18,19]. These components are involved in various biological processes, including human neurodevelopment, learning and memory, osteogenic differentiation (Figure 1 and Figure 2), regulation of cardiovascular homeostasis, and immune regulation, as well as tumor invasion and prognosis [20].

Understanding the mechanisms underlying disease occurrence and treatment enables clinicians to formulate personalized treatment plans tailored to various disease conditions. This approach promotes the advancement of personalized precision medicine, enhances the therapeutic efficacy of bone tissue repair, and mitigates adverse reactions associated with treatment. Therefore, exploring the occurrence and treatment of diseases from the perspective of molecular mechanisms is of utmost importance. A significant body of research has emerged regarding the regulatory role of m6A in the osteogenesis process, which is expected to advance the development of precision medicine. This review specifically highlights the regulatory roles of the three major components of m6A (“Writer”, “Eraser”, and “Reader”) in osteogenesis.

## 2. Methods

A comprehensive search was conducted to investigate the relationship between m6A modification and osteogenic differentiation or bone regeneration. Relevant articles, including reviews, animal and human experimental studies, observational studies, and meta-analyses, were selected from the PubMed and Web of Science databases. Evidence from these publications was systematically extracted, and the overall findings were summarized in a narrative format.

### 2.1. The Relation of m6A to Osteogenesis and Bone Diseases

As previously mentioned, m6A typically consists of three components: m6A methyltransferases, referred to as “Writers” (which mainly include METTL3, METTL14, and WTAP), m6A demethylases, termed “Erasers” (such as FTO and ALKBH5), and m6A binding proteins, known as “Readers” (including YTHDF1/2/3, YTHDC1/2, and IGF2BPs) [17,18,19]. In the maintenance of bone homeostasis, it is essential not only to mediate osteogenic differentiation but also to sustain the balance between osteogenesis and bone resorption by osteoclasts [2,21]. The m6A methylation modification typically upregulates the expression of osteogenic-related factors, promoting osteogenic differentiation [19,22,23,24,25,26,27,28,29,30,31,32,33,34,35,36,37,38]. The deficiency of methylation modification results in a diminished osteogenic differentiation potential, reduced bone mass, and impaired bone formation [22,25,39,40,41,42,43], while concurrently exerting stimulative effects on adipogenic differentiation and osteoclast transformation [44,45,46]. Currently, the primary focus regarding m6A methylation modification pertains to osteoporosis (OP) and bone loss resulting from periodontitis. Numerous studies have demonstrated that the level of m6A modification is reduced in patients with OP, and enhancing m6A modification can alleviate OP [22,25,27,28,44,45,47,48,49,50,51,52,53,54], and it also has certain therapeutic effects in the disease of bone loss caused by periodontitis [46,55,56,57,58,59,60,61]. In some cases of bone defects [23,24], fractures [26,62,63], osteomyelitis [64], steroid-associated osteonecrosis of the femoral head (SONFH) [65], and one case of damage from bisphosphonates [66], m6A modification stimulates new bone formation and promotes healing. In addition, m6A methylation modification may exacerbate the progression of some bone-related diseases, for instance ankylosing spondylitis [67], ossification of the ligamentum flavum [68], chronic nephrosis [69], or cardiovascular calcification disorders [70].

### 2.2. The Role of m6A “Writers” in Osteogenesis

The m6A writer is a protein complex with a molecular weight of approximately 1 Mda [71]. METTL3 is the earliest identified key enzyme involved in m6A methylation modification, which can be detected in both the nucleus and cytoplasm, playing a crucial role in the regulation of m6A [72]. Studies have demonstrated that the knockout of METTL3 directly results in a reduction in m6A levels in mammalian embryonic stem cells, HeLa cells, and HepG2 cells [72]. As the second methyltransferase, METTL14 serves as an excellent adaptor protein that enhances METTL3 activity. METTL14 shares homology with METTL3 [73], and can form a stable heterodimeric core complex with METTL3 [74]; it plays a significant role in B cell survival, insulin secretion, and glucose homeostasis [75]. WTAP is also a critical component of the writer complex, recruiting METTL3 and METTL14 to mRNA targets to collaboratively catalyze the formation of m6A [76]. Depletion of WTAP could induce a loss of nuclear speckle localization for METTL3 and METTL14 [77]. Additionally, proteins such as vir-like m6A methyltransferase-associated, zinc finger CCCH-type containing 13, CBLL1 (also known as HAKAI), RNA-binding motif protein 15/15B, and methyltransferase-like 16 (METTL16) play significant roles in the regulation and modification of m6A methylation [78].

#### 2.2.1. The Role of METTL3 in Osteogenesis

Runt-related transcription factor-2 (Runx-2), a crucial transcription factor during embryonic development, plays a pivotal role in directing the differentiation and development of skeletal cells [79]. Studies have confirmed that METTL3 modified Runx-2 mRNA in bone marrow mesenchymal stem cells (BMSCs) by m6A methylation to maintain its stability, and increased Runx-2 expression to promote bone formation [22,23,24]. Yan et al. constructed an OP model in C57BL/6J mice via ovariectomy (OVX), and findings from in vivo studies showed that the mRNA expressions of METTL3 and METTL14 in bone were significantly reduced. METTL3 led to a decrease in the expression of miR-320, partially modifying Runx-2 through m6A methylation, while maintaining Runx-2 expression at higher levels [22]. Jiao et al. found that β-TCP(A biological scaffold) stimulated METTL3 expression of BMSCs, which in turn increased the m6A level of Runx-2 mRNA, thereby contributing to its stability and level [23]. Zhou et al.’s in vitro studies have proved that METTL3 plays a promoting role in the IGF2BP1/m6A/Runx-2 signaling axis during the osteogenic differentiation of BMSCs [24] (Table 1).

METTL3 also interacts with other osteogenic factors, participating in the regulation of signaling pathways and influencing osteogenic differentiation [25,26,27,28] (Table 1). The parathyroid hormone (PTH)/parathyroid hormone 1 receptor (PTH1R) signaling axis serves as an important downstream pathway for m6A regulation in mouse BMSCs. Notably, it is reported from in vivo studies that when METTL3 is knocked out, the translation efficiency of PTH1R is diminished and METTL3 overexpression can mitigate OP caused by estrogen deficiency in mice [25]. A study in vitro demonstrated that when silencing METTL3, the m6A methylation level of histone deacetylase 5 (HDAC5) in BMSCs is directly reduced, leading to downregulation of HDAC5 mRNA expression, and the expression of osteogenic genes decreases [26]. METTL3 was up-regulated in BMSCs of Sprague Dawley (SD) male rats in vitro, while the expressions of VEGF-a, Runx-2, and osterix decreased following METTL3 knockdown, and alkaline phosphatase (ALP) activity and the formation of mineralized nodules were reduced. Additionally, a significant decrease in protein kinase B (AKT) phosphorylation was observed [27]. Overexpression of METTL3 can activate the Wnt signaling pathway, enhancing the osteogenic potential of OP BMSCs in vitro and promoting in vivo bone tissue healing [28].

Some RNAs lack the ability to code for proteins themselves but can regulate the expression of protein-coding genes by either recruiting or isolating these genes. Long non-coding RNAs (lncRNAs) typically play a role in regulating chromatin remodeling, mRNA degradation, transcription, protein activity, and translation, often functioning as miRNA sponges [80]. Peng et al. demonstrated that METTL3 promotes osteogenic differentiation of human BMSCs by regulating the LINC00657/miR-144-3p/BMPR1B axis in vitro [47]. Li et al. showed that METTL3-mediated methylation of the lncRNA MIR99AHG, which targets miR-4660, enhances the osteogenic differentiation of BMSCs in vitro [48].

In vitro studies have shown that the overexpression of METTL3 increases the m6A methylation level in human periodontal ligament stem cells (PDLSCs), thereby enhancing cells proliferation, osteogenic differentiation, and migratory abilities. Conversely, the inhibition of METTL3 results in a decrease in these biological activities of PDLSCs [29,30]. Sun et al. discovered that METTL3 initiates m6A mRNA methylation, which enhances the stability of Yes-associated protein (YAP) mRNA. Additionally, YTHDF1 and eukaryotic translation initiation factor 3a (eIF3a) are recruited into the translation initiation complex to regulate YAP mRNA translation, thus promoting the osteogenic differentiation of PDLSCs in vitro [31]. Furthermore, Sun demonstrated that METTL3-mediated methylation facilitates Runx-2 expression, promoting the osteogenic differentiation of PDLSCs [32] (Table 1).

METTL3 also has positive effects on other mesenchymal stem cell osteogenesis [19,33,34,35] (Table 1). Some in vitro studies have been reported. Yang et al. demonstrated that METTL3 can activate the Wnt/β-catenin signaling pathway by regulating the m6A modification and expression of lncSNHG7 in human dental pulp stem cells (DPSCs), thereby enhancing their osteogenic and odontogenic differentiation [33]. Cai et al. inhibited METTL3 expression in human DPSCs and observed that both cell proliferation and osteogenic differentiation were impaired. METTL3 directly interacts with ATP citrate lyase (ACLY) and the mitochondrial citrate transporter (SLC25A1), influencing the glycolytic pathway [34]. Additionally, METTL3 indirectly interacts with circCTTN (a circular RNA) through the tumor aggressiveness markers nucleolar protein (NOP2) and eIF3a. An increase in the m6A methylation level of circCTTN was found to promote the osteogenic differentiation of human umbilical cord mesenchymal stem cells (HUC-MSCs) [35]. Moreover, METTL3 can activate the mitogen-activated protein kinase (MAPK) signaling pathway by regulating m6A modification and expression of lncRNA, thereby stimulating the osteogenic differentiation of human adipose-derived mesenchymal stem cells (ADSCs) [19].

#### 2.2.2. Osteogenic Role of METTL3 in the Microenvironment

Inflammation is closely associated with m6A methylation modification [81]. Macrophages typically serve as the initiators of the inflammatory response. In addition to facilitating the onset of inflammation and local microbial clearance, macrophages mediate the interaction between the functions of BMSCs and the skeletal system, playing a significant role in bone formation and remodeling. During the early stages of bone formation, the ability of BMSCs to differentiate into bone cells and migrate to the fracture site is crucial [82,83,84]. Studies have demonstrated that METTL3 enhances the bone differentiation and migration of BMSCs [26].

METTL3 has a close relationship with periodontitis and alveolar bone resorption (Table 1). Some in vitro experiments showed the expression of METTL3 in human PDLSCs is increased, METTL3 is knocked down, the expression of pro-inflammatory cytokines and osteogenic markers in the cells is decreased, the activity of ALP and the formation of mineralized nodules are reduced, and the activation of the phosphatidylinositol 3-kinase (PI3K)/AKT signaling pathway is inhibited [55]. Wang et al. found that forkhead box protein O1 (FOXO1) was generally expressed at a low level in periodontitis patients. FOXO1 can affect the osteogenic differentiation of PDLSCs by regulating METTL3 methylation modification of the PI3K/AKT signaling pathway [56]. Huang et al. revealed the involvement of the lipopolysaccharide (LPS)/METTL3/14–solute carrier family 39 member 9 (SLC39A9)/zinc/IL-6 axis and the p38 and c-Jun N-terminal kinase (JNK)/MAPK signaling pathways in the inflammatory response of PDLSCs [57].

Chen et al. confirmed that METTL3-mediated m6A modification could promote the expression and stability of lncRNA CUTALP in PDLSCs in the periodontitis environment, and lncRNA CUTALP inhibited miR-30b-3p and regulated the expression of Runx-2, thus promoting the osteogenesis of human PDLSCs [58]. Zhang et al. found that METTL3 promoted the osteogenic differentiation of PDLSCs in an inflammatory environment by regulating the stability of lncRNA 4114 and upregulating its expression level, while METTL3 knockdown had an inhibitory effect [59] (Table 1).

METTL3 was upregulated during the osteogenic differentiation of mouse embryo osteoblast precursor cells (MC3T3-E1), activating the Smad-dependent signaling pathway. Additionally, METTL3 plays a role in regulating the MAPK signaling pathway during LPS-induced inflammation, stimulating the phosphorylation of extracellular regulated protein kinase (ERK), p38, JNK, and p65, which activates inflammatory responses [85]. Zhang et al. demonstrated in vitro that in LPS-stimulated osteoblasts, METTL3 overexpression activates the Wnt/β-catenin/c-Myc signaling pathway, enhances ribosomal and mitochondrial functions, and mediates osteoblast behavior. Conversely, METTL3 knockout inhibits ribosome biogenesis and oxidative phosphorylation, leading to the establishment of a mouse model of periodontitis. Furthermore, in vivo METTL3 inhibitors, such as SAH (METTL3 and METTL14 inhibitor), exacerbate periodontal bone loss and local inflammatory states, while the Wnt/β-catenin pathway activator, CHEL-99021 HCl, partially mitigates these bone losses [60].

The endoplasmic reticulum (ER) is a crucial organelle that coordinates nearly all aspects of cellular protein synthesis, folding, and maturation [86]. In vitro studies have demonstrated that METTL3 plays a critical role in maintaining ER homeostasis and influences apoptosis, proliferation, and differentiation of MC3T3-E1 cells. However, severe ER stress causes METTL3 to inhibit the expression of glucose-regulated protein 78 (Grp78) through YTHDF2-mediated mRNA degradation, thereby inducing ER stress and leading to apoptosis and impaired differentiation of MC3T3-E1 cells [87]. The activation of the unfolded protein response (UPR) pathway enables cells to adapt to ER stress and survive, which is essential for osteogenesis [88]. However, continuous activation of the UPR pathway under heightened sensitivity to ER stresses, such as inflammation, calcium ion imbalance, and hypoxia, can disrupt the homeostatic functions of the ER, trigger the apoptotic process, disrupt the homeostatic functions of the ER, and lead to the accumulation of misfolded or unfolded proteins resulting in ER stress [89,90], which may vary under different experimental conditions and cellular environments. The severity of ER stress is not uniform, and its intensity is a significant contributor to the observed outcomes. Given the complexity and adaptability of the in vivo microenvironment, the interplay between METTL3-mediated m6A methylation and ER stress in osteogenesis warrants further investigation.

Studies have found that miR-320a negatively regulates phosphatidylinositol-4,5-bisphosphate 3-kinase catalytic subunit alpha (PIK3CA), which exacerbates the progression of staphylococcal protein A-mediated osteomyelitis (OM), including aspects of osteogenesis, oxidative stress, and the inflammatory response. METTL3 restrains the expression of BMSCs’ miR-320a in an m6A-dependent manner, suppressing the development of OM [64].

#### 2.2.3. Effects of METTL3 Methylation-Related Drugs on Osteogenesis

Exploring mechanisms to develop osteogenic drugs, achieving precision medicine, and reducing the side effects of drugs on the human body are also points that need to be considered in bone tissue repair treatment. Zhang et al.’s in vitro research demonstrated that methotrexate (MTX) impedes the synthesis of S-adenosylmethionine (SAM), leading to decreased levels of m6A methylation and a subsequent reduction in BMSCs’ osteogenic capacity. The addition of betaine, which increases intracellular SAM production, was found to restore the inhibited m6A content and promote osteogenesis in MTX-treated cells. Furthermore, Zhang indicated that the bone-promoting effects of m6A methylation, mediated by single carbon metabolism, may be linked to hypoxia-inducible factor-1α (HIF-1α) and glycolytic pathways. Dimethyloxalylglycine also was shown to recover the osteogenic potential of MTX-treated cells by upregulating the expression of HIF-1α and key glycolytic enzymes [91]. Wang et al. reported that QGD (A Chinese medicine) enhanced BMSCs’ differentiation and mitigated OP induced by OVX in SD rats through METTL3-mediated m6A modification in vitro [92]. Tian et al. treated BMSCs from BALB/c mice with Ecliptae herba extract for a 9-day in vitro osteogenic induction culture, resulting in significant increases in the expression of METTL3 and METTL14; while the expression of WTAP remained unchanged, the activity of ALP and ossification levels were elevated. Knocking out METTL3 led to decreased ALP activity, reduced formation of mineralized nodules, and diminished mRNA expression of bone formation markers such as osterix and osteocalcin. Concurrently, Ecliptae herba was shown to promote the enrichment of HIF-1α, as well as the PI3K/AKT and Hippo signaling pathways in cells, which are associated with m6A modifications [93]. Additionally, as previously mentioned, METTL3 directly interacts with ACLY and SLC25A1, influencing the glycolytic pathway [34]. It is well known that HIF-1α is closely related to glycolysis, and energy supply is a necessary condition for osteogenic differentiation [94]. The relationship between drugs, METTL3, HIF-1α, and energy metabolism needs to be further explored.

#### 2.2.4. Negative Role of METTL3 Methylation Modification in Disease

In some diseases characterized by excessive calcification, METTL3 may exacerbate disease progression (Table 1). Ye et al. demonstrated that METTL3 stimulates the osteogenic differentiation of BMSCs in the tibia via the homeobox D8 (HOXD8)/integrin alpha 5 (ITGA5) axis in cases of congenital pseudo-arthropathy [95]. Zhou et al. demonstrated that METTL3 promotes the osteogenic differentiation of human aortic valve mesenchymal cells (AVMSCs) by inhibiting twist family bHLH transcription factor 1 (TWIST1) through the m6A-YTHDF2-dependent pathway, thereby contributing to the development of cardiac disease associated with aortic valve calcification [96]. Furthermore, Yuan et al. found that METTL3 enhanced the m6A methylation of lncRNA XIST, which plays a role in the ossification of primary ligament fibroblasts through the miR-302a-3p/ubiquitin carboxyl-terminal hydrolase 8 (USP8) axis, suggesting that METTL3 is closely linked to the pathogenesis of ossification of the posterior longitudinal ligament [97].

### 2.3. The Role of METTL14 in Osteogenesis

Some studies have reported that METTL14 has a positive regulatory effect on osteogenesis (Table 1). An in vitro study has shown that METTL14 activates the osteogenic differentiation of BMSCs by stimulating the β-catenin/transcription factor 1 (TCF1)/Runx-2 axis [49]. Huang et al. reported that METTL14 was downregulated in OP patients; the inhibition of METTL14 affected the stability of Smad1 at the protein level, thereby hindering the osteogenic differentiation of human BMSCs in vitro. Conversely, overexpression of METTL14 resulted in increased bone mass in C57BL/6 female OVX mice in vivo [50]. Jin et al. proposed that icariin (ICA) upregulates METTL14, which could maintain the mRNA stability of prolyl 4-hydroxylase subunit beta (P4HB), promote the osteogenic differentiation of human BMSCs in vitro, and restore bone mass in OP female SD-OVX rats in vivo [51]. He et al. demonstrated that METTL14 increased the m6A modification level in beclin-1 mRNA transcripts, thereby triggering the autophagy signaling pathway, with IGF2BPs further maintaining the stability of beclin-1. This process plays a crucial role in regulating the osteogenic differentiation of BMSCs in vitro and impeding the development of OP in C57BL/6J female OVX mice in vivo. Furthermore, METTL14 inhibits the differentiation of macrophages into osteoclasts and reduces bone resorption [52]. Wang et al. indicated that METTL14 can bind to sirtuin 1 (SIRT1) and enhance the stability of SIRT1 mRNA through m6A methylation modification, thereby regulating the osteogenic differentiation of mouse BMSCs, inhibiting osteoclast differentiation of bone marrow mononuclear macrophages (BMMs) in vitro, and alleviating the progression of OP in mice in vivo [53]. Cheng et al. demonstrated that METTL14 upregulates the m6A level of protein tyrosine phosphatase non-receptor type 6 (PTPN6) and enhances the stability of PTPN6 mRNA. This regulation facilitates the interaction between PTPN6 and GSK-3β, promotes the translocation of β-catenin to the nucleus, and accelerates the activation of Wnt signal transduction. These processes stimulate the proliferation and osteogenic differentiation of steroid-associated osteonecrosis of the femoral head (SONFH) BMSCs in vitro [65].

Wu et al. demonstrated that compression force can regulate the level of insulin-like growth factor 1 (IGF1) through mediation by METTL14, which enhances the viability and osteogenic differentiation of PDLSCs in vitro [98]. Xie et al. provided evidence that tumor necrosis factor-α (TNF-α) accelerates ankylosing spondylitis (AS) through the METTL14-dependent m6A modification of engulfment and cell motility protein 1, directing the migration of AS MSCs in vitro [67].

#### 2.3.1. The Role of WTAP in Osteogenesis

In the absence of WTAP, the RNA binding capacity of METTL3 was significantly diminished, indicating that WTAP may play a crucial role in regulating the recruitment of the m6A methyltransferase complex to mRNA targets [76,77]. Some in vitro studies, for example You et al., have shown that WTAP is downregulated in OP patients, upregulated during the osteogenic differentiation of mouse BMSCs, and downregulated during lipogenic differentiation [44]. Liu et al. demonstrated that WTAP-mediated m6A methylation facilitated osteogenic differentiation while inhibiting lipogenic differentiation of mouse BMSCs via the miR-29b-3p/histone deacetylase 4 (HDAC4) axis [45].

circRNA is highly expressed in the nucleus and cytoplasm of eukaryotic cells. It also exerts biological functions through miRNA sponge absorption, transcriptional regulation, interaction with RNA-binding proteins, and translation into peptide form [99,100]. It was found that upregulation of WTAP expression increases m6A modification in circCDK14, which is further recognized by IGF2BP3 and promotes the stability of circCDK14; increased circCDK14 expression further stimulated osteogenic differentiation of ligamentum flavum cells through sponge absorption of the miR-93-5p/ALF transcription elongation factor 4 (AFF4) pathway in vitro [68].

In addition to this, Gao et al. reported that WTAP regulates the mRNA stability of Runx-2 and promotes the osteogenic differentiation of human stem cells from the apical papilla (SCAPs) in vitro [101].

#### 2.3.2. Osteogenic Role of WTAP in the Microenvironment

In the context of the inflammatory microenvironment, WTAP may negatively impact osteogenesis. Li et al. applied LPS to mouse macrophages and BMSCs in vitro, finding that it increased WTAP expression in macrophages, induced by inflammation. The inhibition of WTAP expression facilitated the transition from the M1 to M2 macrophage phenotype; this transition subsequently enhanced the osteogenic differentiation of BMSCs and improved periodontal immunity. In vivo experiments demonstrated that inhibition of WTAP reduced bone loss in a C57BL/6J mouse model of periodontitis [46].

### 2.4. Other Methyl Transfer-like Enzymes Related to Osteogenesis Reports

Biobehavioral disorders associated with suture mesenchyme stem cells (SuMSCs) have resulted in abnormal cranial suture morphology and uncoordinated adaptation to brain development, frequently accompanied by neurocognitive deficits [100]. Research by Lei et al. has demonstrated that the loss of METTL5 leads to skull deformities and compromised cranial sutures. METTL5 is shown to regulate the osteogenic differentiation of C57BL/6J mouse SuMSCs via the Wnt signaling pathway, indicating that methylation modification plays a crucial role in cranial suture development [102]. Jin et al. found that METTL7a regulates m6A modification in vitro and mitigates the impairment of human BMSC function caused by bisphosphonates [66].

**Table 1:** The roles of m6A “Writers” in osteogenesis and the bone microenvironment.

Regulators	Cells and Species	Research Background	Regulators’ Biological Functions and Involved Signaling Pathways	Ref.
METTL3	54 to 65 years OP female, C57BL/6 mice BMSCs, OVX C57BL/6 mice OP model	OP	Human OP bone samples: METTL3 (↓), m6A (↓), METTL3 (↑) → m6A methylation of stability (level) (↑) → osteogenesis (↑) → OP (↓); METTL3 (↑) → m6A methylation of pre-miR-320 (↑) → miR-320 (↓) → Runx-2 stability (level) (↑) → osteogenesis (↑) → OP (↓)	[22]
METTL3	SD male rats BMSCs	Skull defects	β-TCP → METTL3 (↑) → m6A methylation of Runx-2 mRNA stability (level) (↑)	[23]
METTL3	BMSCs	Maxillofacial bone defects	METTL3 (↑) → m6A methylation of Runx-2 mRNA stability (level) (↑)	[24]
METTL3	Mouse BMSCs	OP caused by estrogen deficiency	METTL3 (↑) → m6A methylation of PTH/PTH1R (↑) → osteogenesis (↑), lipogenesis (↓)	[25]
METTL3	Mouse Macrophage Cell line RAW 264.7, C57BL/6 mouse BMSCs	Bone fracture healing (migration and differentiation)	Macrophage cell line RAW 264.7: METTL3 (↑) → m6A methylation of HDAC5 (↑); BMSCs: METTL3 (↑) → m6A methylation of Runx-2 mRNA stability (level) (↑)	[26]
METTL3	SD male rats BMSCs	OP	METTL3 (↓) → m6A methylation (↓) → Akt (↓) → VEGF-a, Runx-2, and osterix (↓)	[27]
METTL3	OP-BMSCs, OVX SD rat OP model	OP	METTL3 (↑) → m6A methylation (↑) → Wnt (↑) → osteogenesis (↑)	[28]
METTL3	Human BMSCs	OP	METTL3 (↑) → m6A methylation of LINC00657 (↑) → miR-144-3p (↓) → BMPR1B (↑) → osteogenesis (↑)	[47]
METTL3	Female C57BL/6J mouse BMSCs	OP	METTL3 (↑) → m6A methylation of lncRNA MIR99AHG → lncRNA MIR99AHG (↓) → miR-4660 (↑) → osteogenesis (↑)	[48]
METTL3	Human PDLSCs	Osteogenic differentiation	METTL3 (↑) → m6A methylation (↑) → proliferation, osteogenic differentiation, migration (↑)	[29,30]
METTL3	Human PDLSCs	Osteogenic differentiation	METTL3 (↑) → m6A methylation (↑) → IGF2BP1(↑) → YAP mRNA stability (level) (↑) → osteogenesis (↑)	[31]
METTL3	Human PDLSCs	Osteogenic differentiation	METTL3 (↑) → m6A methylation (↑) → IGF2BP1 (↑) → Runx-2 mRNA stability (level) (↑) → osteogenesis (↑)	[32]
METTL3	Human DPSCs	Osteogenic/odontogenic differentiation	METTL3 (↑) → m6A methylation of lncSNHG7 → Wnt/β-catenin (↑) → osteogenesis (↑)	[33]
METTL3	Human DPSCs	Osteogenic differentiation	METTL3 (↑) → m6A methylation of ACLY and SLC25A1(↑) → osteogenesis and proliferation (↑); IGF2BP2 and IGF2BP2/3 (↑) → ACLY and SLC25A1 stability (↑)	[34]
METTL3	HUC-MSCs	Osteogenic differentiation	METTL3 (↑) → m6A methylation of circCTTN (↑) → osteogenesis (↑)	[35]
METTL3	Human ADSCs	Osteogenic differentiation	METTL3 (↑) → m6A methylation of lncRNA RP11-44 N12.5 (↑) → MAPK (↑) → osteogenesis (↑)	[19]
METTL3	Human PDLSCs	Periodontitis	Periodontitis → METTL3 (↑), METTL3 (↓) → proinflammatory factor, osteogenesis (↓) and PI3K/Akt (↓)	[55]
METTL3	Human PDLSCs	Periodontitis	FOXO1 → METTL3 (↑) → m6A methylation of PI3K/AKT → osteogenesis (↑)	[56]
METTL3	Human PDLSCs	Periodontitis	LPS → METTL3/14 (↑) → m6A methylation of SLC39A9 → SLC39A9 (↓) → zinc (↓) → IL-6 (↑)	[57]
METTL3	Human PDLSCs	Periodontitis	METTL3 (↑) → m6A methylation of lncRNA CUTALP (↑) → miR-30b-3p (↓) → Runx2 (↑)	[58]
METTL3	Human PDLSCs	Periodontitis	METTL3 (↑) → m6A methylation of IncRNA 4114 (↑) → osteogenesis (↑)	[59]
METTL3	MC3T3-E1	LPS-induced inflammation	METTL3 (↓) → Smad7 and Smurf1 (↑) → osteogenesis (↓), METTL3 (↑) → MAPK (↑) → ERK, p38, JNK, and p65 phosphorylation	[85]
METTL3	MC3T3-E1, Male C57BL/6 mice	Periodontitis	METTL3 (↑) → Wnt/β-catenin/c-Myc → Ribosome and mitochondrial function (↑), METTL3 (↓) → periodontitis was aggravated in mice	[60]
METTL3	MC3T3-E1	ER Stress	METTL3 (↓) → YTHDF2-mediated Grp78 mRNA degradation → apoptosis and differentiation of MC3T3-E1 cells were impaired	[87]
METTL3	Human BMSCs	Osteomyelitis	METTL3 (↑) → m6A methylation of pri-miR-320a → miR-320a (↓) → PIK3CA (↑), regulates osteogenesis, oxidative stress, inflammation	[64]
METTL14	Human BMSCs	OP	METTL3 (↑) → β-catenin (↑) → TCF1 (↑) → Runx-2 (↑) → osteogenesis (↑)	[49]
METTL14	Human BMSCs, OVX-C57BL/6 mice OP model	OP	METTL14 (↓) → Smad1 protein level (↓) → osteogenesis (↓); METTL14 (↑) → C57BL/6 mice having increased bone mass	[50]
METTL14	Human BMSCs, OVX SD rat OP model	OP	ICA → METTL14 (↑) → P4HB mRNA stability (level) (↑) → osteogenesis (↑), restores bone mass in OP rats	[51]
METTL14	C57BL/6 mice-BMSCs, OVX C57BL/6 mice OP model	OP	METTL3 (↑) → m6A methylation of beclin-1, IGF2BPs maintain beclin-1 stability → osteogenesis (↑), prevent the development of OP, and inhibit osteoclast differentiation	[52]
METTL14	Mouse BMSCs, BMMs, OVX C57BL/6 mice OP model	OP	METTL14 (↑) → m6A methylation of SIRT1 → SIRT1 mRNA stability (level) (↑) → regulate the osteogenesis of mouse BMSCs and inhibit osteoclast differentiation of BMMs, alleviating the progression of OP in mice	[53]
METTL14	Human patients BMSCs	Steroid-associated osteonecrosis of the femoral head	METTL14 (↑) → m6A methylation of PTPN6 → PTPN6 mRNA stability (level) (↑) → PTPN6 combine with GSK-3β → Wnt activation → BMSC proliferation and osteogenesis (↑)	[65]
METTL14	Human PDLSCs	Biomechanics	METTL14 → m6A methylation of IGF1 → IGF1 (level) (↑) → osteogenesis (↑)	[98]
METTL14	Human healthy and patients BMSCs	Ankylosing spondylitis	TNF-α → METTL14 → m6A methylation of ELMO1 → BMSC migration (↑), exacerbating the progression of the disease	[67]
WTAP	Mice BMSCs	OP	WTAP → m6A methylation of pri-miR-181a and pri-miR-181c → miR-181a and miR-181c (↑) → SFRP1 (↓) → osteogenesis (↑)	[44]
WTAP	Mice BMSCs	OP	WTAP → m6A methylation of miR-29b-3p → HDAC4 (↓) → osteogenesis (↑), adipogenesis (↓)	[45]
WTAP	Ligamentum Flavum	Ossification of the ligamentum flavum	WTAP → m6A methylation of circCDK14 → IGF2BP3 regulates circCDK14 stability → miR-93-5p (↓) → AFF4 (↑) → osteogenesis (↑)	[68]
WTAP	Human SCAPs	Osteogenic differentiation	WTAP → Runx-2 stability → osteogenesis (↑)	[101]
WTAP	Mice BMSCs and BMMs C57BL/6 mice	Periodontitis	LPS → WTAP (↑) WTAP (↓) → promotes M1 transformation to M2, osteogenesis (↑); WTAP (↓) → decreased bone loss in mice with periodontitis	[46]
METTL5	SuMSCs	Osteogenic differentiation	METTL5 → Wnt → promotes skull and suture development	[102]
METTL7a	Human BMSCs	Bone damage from bisphosphonates	METTL7a → m6A methylation → saves bone damage from bisphosphonates	[66]
METTL3	Human BMSCs	Osteogenic differentiation	METTL3 → m6A methylation of BMP2 → BMP2 mRNA degradation	[103]
METTL3	MC3TCE-1	Osteogenic differentiation and fracture healing	METTL3 → m6A methylation of miR-7212-5p → osteogenesis (↓)	[62]
METTL3	Menstrual blood MSCs	Osteogenic differentiation	METTL3 → m6A methylation of MyD88 → MyD88 (↑) → NF-κB activate osteogenesis (↓)	[104]
METTL14	Human BMSCs	Osteogenic differentiation	METTL14 → m6A methylation of miR-873 → osteogenesis and proliferation (↓)	[105]
m6A	Human ADSCs	Osteogenic differentiation	The total level of m6A decreased with the increase in osteogenic differentiation of ADSCs	[106]
METTL3	Human healthy and peri-implantitis patients	Peri-implantitis	High METTL3 m6A methylation levels were detected in peri-implantitis	[107]
METTL3	Human gingival fibroblasts, male C57BL/6J mice	Periodontitis	METTL3 (↑) → m6A methylation of TNFAIP3 → TNFAIP3 (↓) → decreases the ubiquitination of NEK7 → NLRP3 (↑)	[61]

(↑): enhance, (↓): decrease.

### 2.5. The Role of m6A “Erasers” in Osteogenesis

m6A regulation relies on demethylases, referred to as “Erasers”, including FTO and ALKBH5, which facilitate the demethylation of m6A [78]. FTO, also known as ALKB homolog 9 (ALKBH9) [108], is predominantly located in the nucleus and mediates 5% to 10% of total mRNA m6A demethylation. Additionally, FTO is highly abundant in the cytoplasm of certain leukemia cells, where it can mediate up to 40% of mRNA m6A methylation modification [16]. Studies indicate that FTO knockout can lead to an increase in m6A methylation of intracellular genes [109]. ALKBH5, a homologue of FTO, plays a crucial role in maintaining the balance of m6A modifications within the transcriptome. Unlike FTO, ALKBH5 demethylates by removing methyl groups from m6A-methylated adenosine rather than through oxidation [110]. ALKBH5 inhibits the binding of the reading protein YTHDF2 to plasmacytoma variant translocation 1 (PVT1) by downregulating the m6A modification of PVT1 [111]. The deletion of ALKBH5 has been shown to promote the proliferation, migration, and invasion of pancreatic ductal adenocarcinoma cells both in vivo and in vitro [112].

#### 2.5.1. The Role of FTO in Osteogenesis and the Microenvironment

Several in vitro studies have reported that FTO negatively regulates the osteogenic process (Table 2). Overexpression of FTO in human BMSCs significantly reduced the methylation level of Runx-2 mRNA, accelerated mRNA decay, decreased Runx-2 expression, and inhibited the osteogenic differentiation of human BMSCs [39]. Zheng et al. reported that overexpression of FTO disrupted the stability of miR-7974, increased FK506-binding protein 15 (FKBP15) expression, regulated the recombination of the actin filament system, affected intracellular and extracellular cargo transport, restricted cell remodeling, and inhibited the osteogenic differentiation of human dental follicle stem cells [40].

However, in other in vitro studies the opposite result was manifested (Table 2). FTO promoted the decay of peroxisome proliferative-activated receptor (PPARG) mRNA, which in turn increased the expression of ALP and osteopontin (OPN), thereby promoting bone formation in human BMSCs [113]. Zhang et al.’s in vitro research showed that protein disulfide isomerase family A member 3 (PDIA3) enhanced the osteogenic differentiation of MC3T3-E1 cells, while RNA methylation decreased the stability of PDIA3 mRNA in these cells. FTO was found to reverse the methylation of PDIA3 mRNA. Furthermore, ubiquitin-specific peptidase 20 (USP20) inhibited FTO degradation during the osteogenic differentiation of MC3T3-E1 cells, and PDIA3 increased FTO levels by enhancing USP20 phosphorylation. These findings suggest a positive feedback regulatory loop between PDIA3 and FTO [114].

FTO may play a destructive role in the inflammatory bone microenvironment (Table 2). He et al. found that FTO enhances the mRNA stability and expression of S phase-related proteins (cyclin A2 and CDK2) in osteoclast precursor cells extracted from C57BL/6 mice in a YTHDF2-dependent manner. By promoting cell proliferation and inhibiting apoptosis, the FTO inhibitor FB23-2 reduces osteoclast formation, thereby alleviating bone destruction in mice with periodontitis [115]. Diabetes mellitus (DM) and periodontitis are two prevalent diseases that influence each other. The accumulation of advanced glycation end products (AGEs) due to hyperglycemia may impair cellular function and exacerbate periodontal disease [116,117,118]. Zhou et al. established a disease model of periodontitis with diabetes by employing a high-fat diet combined with streptozotocin injection and silk ligation in C57BL/6 mice. They found that AGEs damage the bone formation of mouse BMSCs in a manner dependent on FTO demethylation [41].

#### 2.5.2. The Role of ALKBH1 and ALKBH5 in Osteogenesis and Microenvironment

There seems to be no consensus on the role of ALKBH5 in osteogenesis (Table 2). Some in vitro studies have shown that ALKBH5 accelerates the degradation of protein arginine N-methyltransferase 6 (PRMT6) mRNA in an m6A-dependent manner, and the ALKBH5/PRMT6 axis regulates the PI3K/AKT pathway, thereby influencing the bone formation of mouse BMSCs [119]. Huang et al. demonstrated that ALKBH5 inhibits the m6A modification of voltage-dependent anion channel 3, leading to accelerated cells senescence and inhibited osteogenic differentiation in OP [42]. However, some in vitro studies showed the opposite; during osteoblast differentiation, both ALKBH5 mRNA and protein expression are upregulated, and ALKBH5 knockdown inhibits osteoblast differentiation and reduces the stability of Runx-2 mRNA [120]. Song et al. demonstrated that lnc-AK311120 promotes the osteogenic differentiation of hADSCs. The knockdown of ALKBH5 significantly increased the m6A modification level of lnc-AK311120 while downregulating its expression [121].

In a type II diabetes peri-inflammatory disease in vivo model using C57BL/6J female mice, it was observed that M1 macrophage polarization increased, while ALKBH5 expression decreased and m6A modification increased, ultimately restricting osteoblast development [122].

ALKBH1 appears to have a positive effect on osteogenesis (Table 2). Cai et al. reported that. the expression of ALKBH1 in mouse BMSCs decreases with aging. The knockout of ALKBH1 enhances lipogenic differentiation while inhibiting osteogenic differentiation in BMSCs in vitro. In vivo experiments further revealed that ALKBH1 knockout mice exhibited decreased bone mass and increased bone marrow obesity [123]. Ouyang et al. found that ALKBH1 facilitates the binding of octamer binding transcription factor 4 (Oct4) to bone morphogenetic protein 2 (BMP2) promoters, thereby activating BMP2 transcription. This process leads to the osteogenic reprogramming of vascular smooth muscle cells (VSMCs) and vascular calcification, contributing to the progression of chronic kidney disease [69].

**Table 2:** The roles of m6A “Erasers” in osteogenesis and the bone microenvironment.

Regulators	Cells and Species	Research Background	Regulators’ Biological Functions and Involved Signaling Pathways	Ref.
FTO	Human BMSCs	Osteogenic differentiation	FTO (↑) → m6A methylation of Runx-2 (↓) → Runx-2 (↓) → osteogenesis (↓)	[39]
FTO	Human BMSCs	OP	FTO (↑) → YTHDF1 → PPARG (↓) → osteogenesis (↑)	[113]
FTO	MC3T3-E1	OP	FTO (↑) → m6A methylation of PDIA3 (↓) → PDIA3 (↑) → osteogenesis (↑), PDIA3 → USP phosphorylation (↑)	[114]
FTO	Human DPSCs	Osteogenic differentiation	FTO (↑) → miR-7974 stability (↓) → FKBP15 (↑) → osteogenesis (↓)	[40]
FTO	Osteoclast precursor cells, C57BL/6 mice	Periodontitis	FTO (↑) → YTHDF2 dependency mode → CDK2 stability (↑) → promotes the proliferation of osteoclasts and inhibits their apoptosis; FTO (↓) → inhibits bone loss in periodontitis	[115]
FTO	C57BL/6 mice BMSCs, C57BL/6 mice	Diabetes mellitus combined with periodontitis	AGEs damage bone formation in an FTO demethylation-dependent manner	[41]
ALKBH5	Mice BMSCs	Osteogenic differentiation	ALKBH5 → m6A methylation of PRMT6 (↓) → PRMT6 (↓) → PI3K/AKT (↓) → osteogenesis (↓)	[119]
ALKBH5	Human BMSCs	OP	ALKBH5 → m6A methylation of VDAC3 (↓) → osteogenesis (↓) and aggravates OP	[42]
ALKBH5	Male embryonic rat-osteoblasts	Osteogenic differentiation	ALKBH5 (↓) → Runx-2 stability (↓) → osteogenesis (↓)	[120]
ALKBH5	Human ADSCs, nude mice mandibular defect model	Osteogenic differentiation	ALKBH5 (↓) → m6A methylation of lnc-AK311120 (↑) → lnc-AK311120 (↓) → osteogenesis (↓)	[121]
ALKBH5	Male C57BL/6J mice	Type II diabetes and peri-implantitis	ALKBH5 (↑) in type II diabetes and peri-implantitis	[122]
ALKBH1	Mice BMSCs, mice	OP	ALKBH1 (↓) → adipogenesis (↑), osteogenesis (↓), loses bone mass and increases fat content	[123]
ALKBH1	Vascular Smooth Muscle Cell	Chronic nephrosis	ALKBH1 → promotes Oct4 combination with BMP2 → angiostenosis	[69]

(↑): enhance, (↓): decrease.

### 2.6. The Role of “Readers” in Osteogenesis

m6A readers are a class of binding proteins that specifically decode the m6A mark and influence methylated mRNAs. They participate in determining mRNA fate by regulating precursor mRNA splicing, promoting translation, controlling mRNA decay, and maintaining protein stability. Different m6A readers serve distinct functions for mRNA [124]. YTH domain proteins, including YTHDF1–3 and YTHDC1–2, represent a significant group of m6A readers [16]. YTHDF1 interacts with initiation factors to enhance translation efficiency [125]. YTHDF2 recognizes and targets m6A-modified mRNA for degradation, thereby regulating mRNA instability [73,126]. YTHDF3 facilitates protein synthesis in conjunction with YTHDF1 while YTHDF2 mediates the decay of methylated mRNA [127]. YTHDC1, located in the nucleus of mammalian somatic cells, regulates mRNA splicing site selection in a concentration-dependent manner [128]. As the largest member of the YTH family, YTHDC2 preferentially binds to the common motif of m6A, enhancing translation efficiency while concurrently reducing the abundance of its target mRNA [18]. Huang et al. reported that IGF2BPs, a group of mRNA binding proteins (including IGF2BP1/2/3), also function as m6A readers for post-transcriptional gene regulation [129]. IGF2BP1, in particular, promotes its function at the translation level in a manner dependent on m6A, stabilizing target mRNAs. Additionally, IGF2BPs regulates various cellular biological processes, including growth, differentiation, and invasion, and its abnormal expression is implicated in the pathological processes of diseases [52].

Several heterogeneous nuclear ribonucleoproteins (hnRNPs), including hnRNPC, hnRNPG, and hnRNPA2/B1, bind to nascent RNA transcripts, influencing the stability, splicing, output, and translation of precursor mRNA [16]. eIF3a can also function as a reader for m6A, directly binding to a single 5′ UTR m6A to facilitate translation under cellular stress [130]. The fragile X mental retardation protein (FMRP) can directly bind to YTHDF2, thereby indirectly maintaining mRNA containing m6A and affecting the nuclear output of m6A-modified RNA targets [131]. Proline-rich coiled-coil 2A (Prrc2a) has been identified as a novel m6A reader that regulates oligodendrocyte expression [131]. In addition to the aforementioned writers, METTL3 can also function as an m6A reader, enhancing translation by binding to a small fraction of cytoplasmic m6A-modified mRNA [132]. Furthermore, some researchers have identified leucine-rich pentatricopeptide repeat-containing protein (LRPPRC) as a potential m6A reader [78,133].

#### 2.6.1. The Role of YTHDF in Osteogenesis

Zinc protein ZNF839 is post-transcriptionally regulated by YTHDF1 in an m6A-dependent manner, interacting with the primary transcription factor Runx-2 to enhance bone formation in human BMSCs in vitro [36]. Gao et al. demonstrated that YTHDF1 enhances the proliferation and osteogenic differentiation of BMSCs in male SD rats in vitro through autophagy and β-catenin pathways [37]. Shi et al. observed that the upregulation of YTHDF1 under hypoxic stress promotes the osteogenic differentiation of MC3T3-E1 cells in vitro, while thrombospondin-1 mRNAs undergo specific methylation modifications to maintain mRNA stability and facilitate in vivo bone formation [38]. Zhang et al. showed that YTHDC1 interacts with HUR (an RNA-binding protein)to enhance the stability of PTPN6 mRNA, thereby positively regulating the expression of PTPN6. The inhibition of PTPN6 promotes osteoclast differentiation and reverses the overexpression of either HUR or YTHDC1 in vitro, contributing to the regulation of OP progression in OVX mice [54] (Table 3).

Ma et al. demonstrated that YTHDC2 binds to Runx-2 mRNA and reduces its intracellular levels, negatively regulating the osteogenic differentiation of rat BMSCs in vitro [134]. Fibulin-1 (FBLN1) mRNA is methylated to increase its stability and promote the bone differentiation of HUC-MSCs. YTHDF2 can induce FBLN1 mRNA degradation, thereby inhibiting osteogenic differentiation, while miR-615-3p further suppresses osteogenic differentiation by regulating YTHDF2 through the m6A-miRNA regulatory mechanism in vitro [43]. In addition, Yuan et al. found that miR-27a negatively regulates the expression of YTHDF2, playing a protective role in SONFH [135].

Feng et al. found that the level of YTHDF3 in the femoral head tissue of postmenopausal osteoporosis (PMOP) patients was significantly reduced. Furthermore, the inhibition of YTHDF3 severely impedes the proliferation and osteogenic differentiation of OP BMSCs in vitro [136].

Zhu et al. provided evidence that Quercetin increases the expression levels of m6A modification (YTHDC, YTHDF) and the Period1 gene in orofacial mesenchymal stem cells (OMSCs), influencing their osteoblastic development in vitro [137].

#### 2.6.2. Osteogenic Role of IGF2BP2 in the Microenvironment

Deepika et al. reported that the levels of IGF2BP1 mRNA and protein were significantly elevated in vitro in the periodontitis group compared to the healthy group. Additionally, Porphyromonas gingivalis LPSs were shown to induce human gingival fibroblasts (HGFs), highlighting the IL-17 signaling pathway as a critical mediator of inflammation [138].

IGF2BP2 plays a dual regulatory role in periodontitis, exhibiting distinct expression patterns in the early and late stages of the disease. Inhibition of IGF2BP2 results in an increased release of inflammatory cytokines, exacerbating early periodontitis. Conversely, in the later stages of periodontitis, IGF2BP2 interacts with CD5L and CD36 mRNA, which negatively impacts the recovery from the disease [139]. Xian et al. found that circHIPK3 interacts with IGF2BP2 in aortic valve interstitial cells (AVICs) to promote kremen1 expression and inhibit the activation of Wnt signal transduction. Furthermore, miR-182-5p plays a role in regulating Wnt signaling by suppressing the expression of Dickkopf, a ligand for Krm1, which is significantly relevant to the development of calcific aortic valve disease (CAVD) [70].

Furthermore, Lai et al. demonstrated that IGF2BP3 enhances the maturation of miR-23a-3p through m6A modification, thereby inhibiting Smad5 and delaying the osteogenic differentiation in MC3T3-E1 cells in vitro and in vivo fracture healing processes [63].

**Table 3:** The roles of m6A “Readers” in osteogenesis and the bone microenvironment.

Regulators	Cells and Species	Research Background	Regulators’ Biological Functions and Involved Signaling Pathways	Ref.
YTHDF1	Human BMSCs	Osteogenic differentiation	YTHDF1 → m6A methylation of ZNF839 → ZNF839 interacts with Runx-2 → osteogenesis (↑)	[36]
YTHDF1	Male SD rat BMSCs	Osteogenic differentiation	YTHDF1 → autophagy and β-catenin → proliferation and osteogenesis (↑)	[37]
YTHDF1	MC3T3-E1	Osteogenesis under hypoxic conditions	YTHDF1 → m6A methylation of THBS1 → THBS stability (↑) → osteogenesis (↑)	[38]
YTHDC1	Mice osteoclasts	OP	YTHDC1 → PTPN6 mRNA stability (↑) → osteoclasis (↓), regulates the process of OP	[54]
YTHDC2	Rat BMSCs	Osteogenic differentiation	YTHDC2 → Runx-2 (↓) → osteogenesis (↓)	[134]
YTHDF2	HUC-MSCs	Osteogenic differentiation	miR-615-3p → YTHDF2 → m6A methylation of FBLN1 (↓) → FBLN1 (↓) → osteogenesis (↓)	[43]
YTHDF2	Human BMSCs	SONFH	miR-27a → YTHDF2 (↓) → alleviates SONFH	[135]
YTHDF3	Human BMSCs	OP	YTHDF3 level (↓) in OP	[136]
YTHDC/YTHDF	Orofacial MSCs	Periodontal bone defects	Quercetin → YTHDC/YTHDF → Period1 (↓) → osteogenesis (↑)	[137]
IGF2BP1	HGFs	Periodontitis	IGF2BP, IL-17 (↑) in periodontitis	[138]
IGF2BP2	Mice BMSCs Mice	Periodontitis	IGF2BP2 controls early periodontitis development, exacerbates advanced periodontitis	[139]
IGF2BP2	Ortica valve interstitial cells	Calcific aortic valve disease	IGF2BP2 interact with circHIPK3 → Kremen1 (↑) → Wnt (↓)	[70]
IGF2BP3	MC3T3-E1	Fracture healing	IGF2BP3 → m6A methylation of miR-23a-3p → Smad5 (↓) → osteogenesis (↓) → delayed fracture healing	[63]

(↑): enhance, (↓): decrease.

## 3. Limitations and Perspectives

However, the impact of m6A methylation modification on osteogenesis remains undetermined. Liu et al. found that METTL3 mediates the methylation of BMP2, leading to the degradation of BMP2 mRNA in BMSCs in vitro [103]. A study shows that METTL3 inhibits the differentiation of MC3T3-E1 cells in vitro by methylating miR-7212-5p [62]. Furthermore, Yu et al. demonstrated that METTL3 regulates the m6A methylation state of MyD88 RNA and positively influences MyD88 expression, which activates the NF-κB signaling pathway and inhibits the osteogenic differentiation of mesenchymal stem cells (MSCs) in vitro [104].

The changes in METTL3 and METTL14 are typically homologous and exhibit a synergistic effect. Additionally, some studies have indicated that the direction of change for METTL14 is opposite to that of METTL3 [62]. Dong et al. found that METTL14 promotes the maturation of miR-873 and inhibits the proliferation and differentiation of human BMSCs through m6A methylation modification in vitro [105]. Sun et al. reported that the overall m6A level decreases during the osteogenic differentiation of ADSCs in vitro [106].

In diverse microenvironments, Burra Anand et al. demonstrated that METTL3 mediates m6A modification in human gingival fibrocytes within the periodontitis, leading to an increased expression of pro-inflammatory factors such as triggering receptor expressed on myeloid cells-1 (TREM-1), interleukin (IL)-1β, and IL-6, which promote the progression of periodontitis [140]. Zhou et al. discovered that the inhibition of METTL3 could suppress NLR family pyrin domain-containing protein 3 (NLRP3) inflammatory-driven pyroptosis, thereby reducing periodontal destruction [61]. Research has indicated that elevated levels of METTL3 m6A methylation were observed in the peri-implant group [107]. This could be due to the implant lacking a periodontal membrane, so that the gum is in direct contact with the alveolar bone, resulting in significant changes to its immune microenvironment. In addition, as mentioned above, the role of FTO and ALKBH5 in osteogenesis is also controversial.

The varying results observed may stem from distinct gene expression patterns and epigenetic modifications influenced by the microenvironments created by different cell origins or disease backgrounds. These variations can lead to the development of cells exhibiting diverse characteristics, such as dryness, proliferation capacity, multilineage differentiation potential, longevity, and immunomodulatory properties. Consequently, these differences can result in varied biological effects, highlighting the specific influences of m6A modification on different targets and environments [106]. Furthermore, discrepancies in results may also arise from the differing inhibition efficiencies of various genes [141].

Furthermore, certain drugs used to treat bone tissue-related diseases, such as OP and osteonecrosis, exhibit adverse reactions that compromise the safety and efficacy of long-term use. Anabolic drugs, including teriparatide, promote bone formation but also increase the risk of coronary heart disease, stroke, diabetes, and osteosarcoma. Additionally, prolonged use of bisphosphonates may elevate the risk of jaw necrosis by inhibiting the normal repair of bone microdamage. Therefore, it is crucial and urgent to gain a deeper understanding of the disease’s pathogenesis at the molecular level to develop viable therapeutic approaches for clinical practice [142,143,144]. Furthermore, the diagnosis of certain bone diseases, such as osteonecrosis of the femoral head, primarily relies on imaging assessments of the femoral head’s blood supply to predict the risk of osteonecrosis. However, because early imaging changes in osteonecrosis of the femoral head are often subtle, clinical diagnosis frequently misses the optimal treatment window [135]. Thus, adopting a molecular network perspective may facilitate the development of new diagnostic methods and enhance the accuracy of disease prediction, ultimately providing fresh insights for treatment strategies.

## 4. Conclusions

Overall, the impact of methylation on osteogenesis may be influenced by factors such as cell type, modeling method, disease state, and other potential regulatory elements. Additionally, m6A can regulate various target genes within the same disease, which presents challenges to the accuracy and safety of drug development. Therefore, the prospects for its development warrant further investigation.

## Data Availability

This research did not generate any new data.

## Abbreviations

The following abbreviations are used in this manuscript:

m6A	N6-methyl adenosine
m1A	N1-methyladenosine
m5C	5-methylcytosine
m7G	7-methylguanine
METTL3	methyltransferase-like protein 3
METTL14	methyltransferase-like protein 14
WTAP	Wilms’ tumor 1-associated protein
FTO	fat mass and obesity-associated protein
ALKBH5	ALKB homolog 5
YTH	YT521-B homology
YTHDF1–3	YTH domain family 1–3
YTHDC1–2	YTH domain-containing proteins 1–2
IGF2BPs	insulin-like growth factor 2 mRNA-binding proteins
METTL16	methyltransferase-like protein 16
BMSCs	bone marrow mesenchymal stem cells
Runx-2	runt-related transcription factor-2
OP	osteoporosis
OVX	ovariectomy
PTH	parathyroid hormone
PTH1R	parathyroid hormone 1 receptor
HDAC5	histone deacetylase 5
SD	Sprague Dawley
VEGF	vascular endothelial growth factor
ALP	alkaline phosphatase
AKT	protein kinase B
LncRNAs	long non-coding RNAs
PDLSCs	periodontal ligament stem cells
YAP	Yes-associated protein
eIF3a	eukaryotic translation initiation factor 3a
DPSCs	dental pulp stem cells
ACLY	ATP citrate lyase
NOP2	nucleolar protein 2
HUC-MSCs	human umbilical cord mesenchymal stem cells
MAPK	mitogen-activated protein kinase
ADSCs	adipose-derived mesenchymal stem cells
PI3K	phosphatidylinositol 3-kinase
FOXO1	forkhead box protein O1
LPS	lipopolysaccharide
SLC39A9	solute carrier family 39 member 9
JNK	c-Jun N-terminal kinase
MC3T3-E1	mouse embryo osteoblast precursor cells
ERK	extracellular regulated protein kinase
ER	endoplasmic reticulum
Grp78	glucose-regulated protein 78
UPR	unfolded protein response
PIK3CA	phosphatidylinositol-4,5-bisphosphate 3-kinase catalytic subunit alpha
OM	osteomyelitis
MTX	methotrexate
SAM	S-adenosylmethionine
HIF-1α	hypoxia-inducible factor-1α
SLC25A1	mitochondrial citrate transporter
HOXD8	homeobox D8
ITGA5	integrin alpha 5
AVMSCs	aortic valve mesenchymal cells
TWIST1	twist family bHLH transcription factor 1
USP8	ubiquitin carboxyl-terminal hydrolase 8
ICA	icariin
TCF1	transcription factor 1
P4HB	prolyl 4-hydroxylase subunit beta
SIRT1	sirtuin 1
BMMs	bone marrow mononuclear macrophages
PTPN6	protein tyrosine phosphatase non-receptor type 6
SONFH	steroid-associated osteonecrosis of the femoral head
IGF1	insulin-like growth factor 1
TNF-α	tumor necrosis factor-α
AS	ankylosing spondylitis
HDAC4	histone deacetylase 4
AFF4	ALF transcription elongation factor 4
SCAPs	stem cells from apical papilla
SuMSCs	suture mesenchyme stem cells
ALKBH9	ALKB homolog 9
PVT1	plasmacytoma variant translocation 1
FKBP15	FK506-binding protein 15
PPARG	peroxisome proliferative-activated receptor
PDIA3	protein disulfide isomerase family A member 3
USP20	ubiquitin-specific peptidase 20
OPN	osteopontin
DM	diabetes mellitus
AGEs	advanced glycation end products
PRMT6	protein arginine N-methyltransferase 6
Oct4	octamer binding transcription factor 4
BMP2	bone morphogenetic protein 2
VSMCs	vascular smooth muscle cells
hnRNPs	heterogeneous nuclear ribonucleoproteins
FMRP	fragile X mental retardation protein
Prrc2a	proline-rich coiled-coil 2A
LRPPRC	leucine-rich pentatricopeptide repeat-containing protein
FBLN1	fibulin-1
PMOP	postmenopausal osteoporosis
OMSCs	orofacial mesenchymal stem cells
HGF	human gingival fibroblasts
AVICs	aortic valve interstitial cells
CAVD	calcific aortic valve disease
MenSCs	mesenchymal stem cells
TREM-1	triggering receptor expressed on myeloid cells-1
IL	interleukin
NLRP3	NLR family pyrin domain-containing protein 3
